# Cresp^®^: transforming the landscape of chemotherapy-induced anemia - a comprehensive retrospective real-world analysis in 523 Indian patients

**DOI:** 10.3389/fonc.2025.1418327

**Published:** 2025-01-24

**Authors:** MV Chandrakanth, Vivek Agarwala, Pranav Sopory, Himadri Nayak, Purvish M. Parikh, Minakshi Roy, Rajib De, Pradeep Narayan, Anjuli Tuladhar Barai, Kaustav Mandal, Moinak Basu, Subhabrata Kumar, Rajdeep Singh Uppal, Syed Mujtaba Hussain Naqvi, Rohit Desai

**Affiliations:** ^1^ Department of Medical Oncology, Narayana Hospital, Kolkata, India; ^2^ Oncology Cluster, Dr Reddy’s Laboratories, Hyderabad, India; ^3^ Department of Medical Oncology, Rabindranath Tagore Hospital, Kolkata, India; ^4^ Department of Clinical Hematology, Mahatma Gandhi Medical College & Hospital, Jaipur, India; ^5^ Hematology Cluster, Dr Reddy’s Laboratories, Hyderabad, India

**Keywords:** chemotherapy-induced anemia, cancer-related anemia, darbepoetin alfa, palliative cancer care, supportive cancer care, retrospective analysis

## Abstract

**Introduction:**

Anemia, a frequently encountered issue among cancer patients undergoing chemotherapy, is regrettably underappreciated despite its prevalence and profound impact on their well-being. Chemotherapy-induced anemia (CIA) diminishes the quality of life, causing fatigue, breathlessness, and a decline in the performance status. However, correcting anemia can lead to notable improvements in these parameters. Notably, darbepoetin alpha (DA) has shown efficacy in addressing anemia in this context. This real-world study aims to evaluate the efficacy of DA in the treatment of CIA among Indian cancer patients.

**Methods:**

This single-center retrospective study assessed the effectiveness of DA in treating CIA among advanced/metastatic solid tumor patients on palliative myelosuppressive therapy. The study measured the change in hemoglobin levels after DA administration as the primary outcome, with secondary outcomes including impact on blood transfusion dependence, changes in anemia-related symptoms, and occurrence of adverse events.

**Results:**

A total of 523 patients, with a median age of 55, were included in the study. Patients were categorized based on cancer site, type of chemotherapy, response to therapy, and DA doses. A significant mean increase of 2.28 gm/dl in hemoglobin (Hb) levels from baseline to post-DA administration was observed (8.56±0.45 to 10.84±0.92; 26.6%; *P<.001*). Each sub-group revealed a significant enhancement of mean hemoglobin from baseline to the end of treatment. Significant improvements were noted from baseline in fatigue, and dyspnea. Adverse drug reactions included hypertension (5.4%), deep vein thrombosis (2.9%), and arrhythmias (0.8%).

**Discussion:**

DA demonstrates impressive efficacy and safety in managing CIA, leading to substantial improvements in mean hemoglobin levels in palliative setting. This has the potential to reduce the need for blood transfusions and enhance the quality of life for patients.

## 1 Introduction

Chemotherapy-induced anemia (CIA) is a common and persistent abnormality in oncology patients (1). While the adoption of targeted therapies (small molecules and biologics) has significantly increased in clinical practice, myelosuppressive chemotherapy remains the backbone of cancer management, especially in low- and middle – income countries. Incidence of CIA is expected to rise in India with the rising incidence in cancer cases (2).

Myelosuppressive chemotherapy directly harms erythroid progenitor cells, whereas the causes of cancer-related anemia (CRA) are more complex. CRA involves inflammatory cytokines, especially IL-6 which leads to functional iron deficiency (FID). FID lowers the production of erythropoietin, which is important for red blood cell growth and development. Hence, the process of erythropoiesis is impaired, which is a key feature of CRA (3).

In clinical practice, CIA is managed primarily via repeated blood transfusions (BT), intravenous (IV) iron or erythropoietin-stimulating agents (ESA) (3, 4). Darbepoetin alfa is approved for treating CIA arising from myelosuppressive chemotherapy in a palliative setting (5). A retrospective analysis of electronic health records from a single center was conducted to assess the real-world effectiveness and tolerability of Cresp^®^ (Dr Reddy’s Laboratories; Darbepoetin alfa) in CIA.

## 2 Materials and methods

### 2.1 Study design and patients

Electronic health records from the department of medical oncology at Narayana Super-specialty Hospital – Howrah, a tertiary care center in Kolkata (West Bengal, India) were accessed from 01/09/2017 to 31/08/2023 (6 years). Inclusion criteria included adult patients (≥18 years), of either gender with a diagnosis of metastatic or advanced solid tumor at the center, completion of at least two cycles of myelosuppressive chemotherapy-based treatment in a palliative setting, and a clinical and/or laboratory diagnosis of CIA with a Hemoglobin (Hb) level of ≤10.0 g/dL. Patients with Hb level of 10.0 - 11.0 g/dL were also considered if they were symptomatic for anemia. Patients who received at least a single dose of Cresp^®^ (200 mcg, s.c., b.i.w.) were included in the study.

Anemia diagnosis was aligned with the Common Terminology Criteria for Adverse Events and the WHO Definition and Criteria for grading cancer-related anemia (CRA) (6). The patient charts were reviewed, and the following information was extracted: demographic data, diagnosis, comorbidities, type of chemotherapy, number of cycles of chemotherapy, response to chemotherapy, baseline Hb levels, baseline requirement of blood transfusion (BT), time of receipt, number of Cresp^®^ doses administered, patient-reported outcomes (PRO), occurrence of treatment emergent adverse events (TEAE). The baseline Hb level was defined as the Hb level (g/dL) at the time of diagnosis of CIA. End of treatment Hb was the last recorded measurement of hemoglobin before the data cut-off. The baseline blood transfusion requirement was defined as per the NCCN guidelines (7). Response to therapy was evaluated as per revised RECIST v1.1 criterion (8).

The exclusion criteria included pediatric patients, early-stage solid-tumor cancers treated with curative intent, hematological malignancies, the use of targeted agents (small molecules and biologics) as monotherapy or in combination with other non-myelosuppressive therapies, and prior exposure to recombinant erythropoietin.

### 2.2 Outcome measures

This study aimed to assess the effectiveness and tolerability of Cresp^®^. Effectiveness parameters included an increase in Hb levels, independence from blood transfusions, and improvement in patient-reported outcomes (PRO). PROs included dyspnea, headache and fatigue as the categories. PROs were evaluated by use of an in-house developed scale which scored each category from 0 to 3. The mean of each score for each category was evaluated at baseline and end of treatment (see **Supplementary Table S1** for details). Tolerability parameters included the occurrence of treatment emergent adverse events (TEAE).

### 2.3 Statistical analysis

A dataset comprising information from the electronic health records of 523 patients was collected for analysis. Baseline demographic variables and other qualitative factors were assessed through frequency percentage tables. Hb increments in the whole cohort and in each sub-group were evaluated using a paired t-test, with a P-value less than 0.05 considered statistically significant. The analysis of Hb increments between sub-groups utilized a one-way ANOVA test. For gradation data, a Wilcoxon Signed Rank Test was conducted to compare baseline and end-of-treatment values.

## 3 Results

### 3.1 Patient characteristics

A total of 523 patients met the inclusion criteria. The median age of the patients was 55 years, with similar distribution between male (50.5%) and female (49.5%) patients. At baseline 98.5% patients had Hb level between 8.0 – 10.0 g/dL, 1.3% had Hb level between 10.0 – 11.0 g/dL, and only one patient had Hb level <8 g/dL. Hypertension was found in 34.3% of patients, diabetes in 17.7%, and chronic obstructive pulmonary disease in 16.2%. The cancer types were classified into seven major groups: gastrointestinal (GI), head and neck squamous cell carcinoma (HNSCC), lung, breast, genitourinary, gynaecological, and others, with GI malignancy being the most prevalent at 31.9%, followed by breast malignancy at 16.3%. Treatment regimens for myelosuppressive chemotherapy were classified either as a single-agent or in combination regimens (multiple-agents), with the majority (74.6%) receiving multiple agents. The chemotherapy protocols were categorized into nine group: taxane + platinum, gemcitabine + platinum, fluoropyrimidine – based, single-agent taxane, platinum + other agent/platinum mono, taxane + other agent, pemetrexed/methotrexate–based, anthracycline–based, and others, with the taxane + platinum group being the most common at 22.9%, followed by gemcitabine + platinum at 18.2%. Chemotherapy regimens were further categorized based on the use of platinum agents as platinum-regimens and non-platinum regimens, with platinum-regimens being more frequently prescribed at 67.7%.

Response to therapy, evaluated according to the RECIST v1.1 criterion for solid tumors and classified as partial response, stable disease, or progressive disease, revealed partial response being the most common outcome in 58.7% of patients, followed by stable disease in 30.4% of patients. Cresp^®^ was administered at a dose of 200 mcg every two weeks, with the number of doses ranging from 4 to 8, and 8 doses being the most frequently administered dose in 81.8% of patients, followed by 4 doses in 11.3% of patients. Detailed patient characteristics are provided **Supplementary Tables S2**-**S6**.

### 3.2 Real – world effectiveness

**Figure 1:**
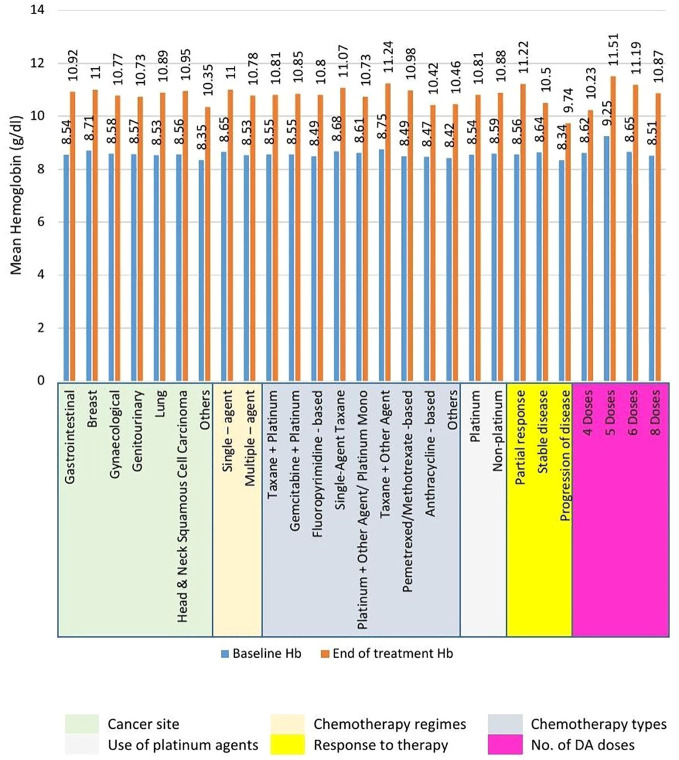
Mean increment in hemoglobin (Hb) levels across subgroups from baseline to end of treatment. Statistically significant (*P<.001*) increment in mean Hb levels was recorded across all subgroups.

The use of Cresp^®^ in managing CIA resulted in a significant increase in the mean Hb level from baseline (8.56 ± 0.45 g/dL to 10.84 ± 0.92 g/dL). This 2.28 g/dL rise in Hb was found to be statistically significant (*P<.001*). Significant improvements (*P<.001*) in mean Hb levels were also observed in all the subgroups between the baseline and the end of treatment levels as shown in **Figure 1**. Analysis between the sub-groups based on therapy response and number of Cresp^®^ doses demonstrated significant improvements in mean Hb levels. According to response to therapy, patients exhibiting partial response had higher improvement in mean Hb levels from baseline at 2.66 ± 0.51 g/dL compared to patients with stable disease (1.86 ± 0.82 g/dL), and disease progression (1.40 ± 0.59 g/dL). Further, patients who received more than 4 doses of Cresp^®^ had higher increment in Hb levels from baseline (5 doses, 2.26 ± 0.54 g/dL; 6 doses, 2.54 ± 1.11 g/dL; 8 doses, 2.36 ± 0.76 g/dL) than patients who received 4 doses of Cresp^®^ (1.61 ± 0.67 g/dL) (see **Supplementary Table S7**).

At baseline, 50 patients (9.6%) required blood transfusion (BL-BT-Cohort). Since Cresp^®^ was administered to all patients, only 22 patients from this cohort ended up receiving blood transfusion (BT). Hence, Cresp^®^ prevented BT in 28 out of 50 patients (risk reduction of 56%) as shown in **Table 1**. Overall, 31 patients (5.9%) in the entire patient pool received BT over the course of their treatment.

**Table 1:** Independence from blood transfusion.

Subgroup	No. of patients	Percentage
Patients requiring Blood Transfusion (BT) at Baseline (BL-BT-Cohort)	50	9.6% of overall patient pool
BL-BT-Cohort receiving BT	22	44% of BL-BT-Cohort
Risk Reduction in BL-BT-Cohort	(28/50)*100	56%

**Figure 2:**
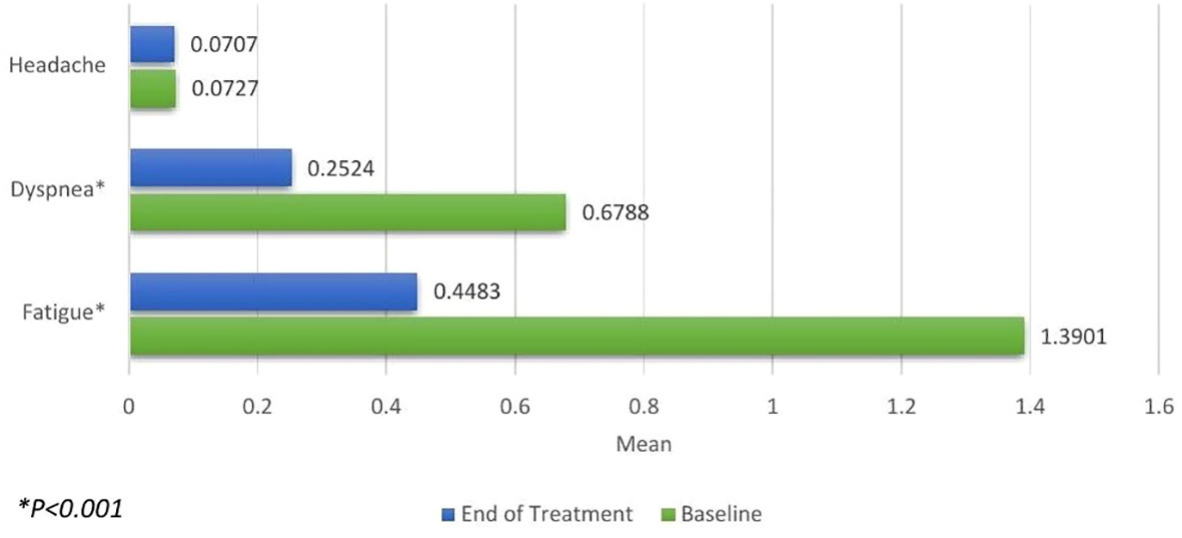
Patient-reported Outcomes for headache, dyspnea and fatigue measured at baseline (green) and the end of treatment (blue). Significant improvements were seen in reduction of dyspnea and fatigue.

Changes in anemia-related symptoms, as per patient-reported outcomes (PRO), were calculated. A significant improvement was observed in the reduction of fatigue (0.9418, *P<.001*) and dyspnea (0.4264, *P<.001*) scales. However, the reduction in the headache scale was not found to be significant (0.0019, *P=0.866*) as shown in **Figure 2**.

### 3.3 Real – world tolerability

Tolerability parameters included the occurrence of treatment-emergent adverse events (TEAE) such as hypertension, hypotension, deep vein thrombosis, arrhythmia, and congestive cardiac failure. A total of 49 patients (9.5%) experienced TEAEs with hypertension being the most common TEAE encountered in 5.4% of patients, followed by deep vein thrombosis in 2.9% of patients, as indicated in **Table 2**.

**Table 2:** Incidence of treatment emergent adverse events.

Adverse Drug Reactions	Incidence	
	No of Patients	Percentage
Hypertension	28	5.4%
Deep vein thrombosis	15	2.9%
Arrhythmia	4	0.8%
Congestive cardiac failure	1	0.2%
Hypotension	1	0.2%
**Total**	**49**	**9.5%**

## 4 Discussion

Epidemiological data in India is limited. Among the top 7 developed countries, the incidence of chemotherapy-induced anemia (CIA) reached as high as 1.6 million patients in 2021 (9). CIA presents symptomatic consequences including poor performance status, fatigue, impaired cognitive function, and exacerbation of existing comorbidities. This compromise in quality of life, combined with decreased drug tolerance (such as chemotherapy dose reduction and delays) and impaired treatment response, contributes to faster tumor progression and shorter overall survival.

Myelosuppressive chemotherapy directly harms erythroid progenitor cells, leading to CIA. On the other hand, the causes of cancer-related anemia (CRA) are more complex. In CRA, tumor and macrophage-produced inflammatory cytokines, especially IL-6, stimulate the liver to produce hepcidin. This hepcidin production results in functional iron deficiency (FID), which is typical in CRA. Functional iron deficiency lowers hypoxia-inducible factor levels, reducing the production of erythropoietin. Without enough erythropoietin, the process of erythropoiesis (red blood cell production) is impaired, which is a key feature of CRA. Additionally, activated macrophages seen in cancer can also make hepcidin. Erythropoietin is crucial for the final maturation of red blood cells and supports their growth and development (3).

While blood transfusion (BT) results in a rapid increase in hemoglobin (Hb) levels, its therapeutic effect is purely symptomatic. However, BT is accompanied by a range of complications, including an independent association with an elevated risk of venous thromboembolism (10). Additionally, transfusion-transmitted infections can disrupt the normal treatment course for a myelosuppressed (neutropenic) patient. Severe complications such as transfusion-related acute lung injury, transfusion-associated circulatory overload, and hemolytic transfusion reactions pose immediate and critical threats (11). The regular need for BTs contributes to an increased overall cost of therapy. Moreover, hospital admissions and the continuous supply of packed red blood cells to blood banks through voluntary donations by relatives and friends are crucial components of the treatment process.

The challenges in managing CIA have led to the development of safe and effective treatments, such as erythropoiesis-stimulating agents (ESAs) recommended in guidelines by NCCN, ESMO, EORTC, and ASCO (7, 12–14). The guidelines vary in their recommendations for initiating and discontinuing ESAs, reflecting differences in the underlying disease and symptoms of individual CIA patients. Meta-analyses have shown the superiority of longer-acting ESAs like darbepoetin alfa over shorter-acting forms such as epoetin (15). A randomized, double-blind, placebo-controlled phase III study focusing on the long-term safety and efficacy of darbepoetin alfa for CIA in patients with advanced non-small cell lung cancer (NSCLC) demonstrated that darbepoetin alfa, dosed to a hemoglobin ceiling of 12.0 g/dL, was non-inferior to placebo in terms of overall survival and progression-free survival. However, it is to be noted that darbepoetin alfa significantly reduced the chances of requiring blood transfusions or experiencing hemoglobin levels equal to or less than 8.0 g/dL in anemic NSCLC patients undergoing myelosuppressive chemotherapy (16).

In our study, a mean increase of 2.28 g/dL in hemoglobin levels was noted, consistent with findings from existing literature (17–19). Subgroup analyses indicated a significant enhancement in hemoglobin levels from baseline to the end of treatment across all subgroups, regardless of cancer site, chemotherapy regimen, no. of doses of Cresp^®^ received or treatment response. Analysis between the sub-groups revealed significant better efficacy in patients taking >4 doses of CRESP^®^. Similar findings were noted in patients exhibiting partial response vs patients having stable disease and/or progressive disease. Furthermore, additional analysis revealed that CRESP^®^ markedly decreased the requirement for blood transfusions in cancer patients, aligning with results from previous clinical investigations (16–19). A small percentage of patients (5.9%) exhibited resistance to the treatment. Significant improvements in anemia-related symptoms, such as fatigue and dyspnea, were observed, with fatigue serving as a key indicator of enhanced quality of life in cancer patients. Multiple studies consistently highlighted substantial improvements in fatigue scores with darbepoetin alfa when compared to placebo (20–22).

In a study conducted by Esquerdo et al., patients were administered darbepoetin alfa at a dosage of 500 mcg once every three weeks during chemotherapy. Significant changes in the mean scores of various Health-Related Quality of Life (HRQoL) measures were noted between baseline and final assessments. The fatigue assessment was conducted using the FACT-F and FSI questionnaires, revealing a substantial difference in mean scores from baseline to final evaluations in patients who achieved an increase in hemoglobin levels exceeding 2 g/dL (23). This study also addressed the risks of treatment-emergent adverse events (TEAE) and concluded that darbepoetin alfa is safe and well-tolerated in cancer patients undergoing chemotherapy. The study by Gascón et al. emphasized a similar safety profile of darbepoetin alfa, with adverse events primarily including hypersensitivity reactions, embolic events, and thrombotic events (16). In a retrospective study by Jagiello et al., adverse events associated with darbepoetin alfa were found to be rare (24). The majority of patients (81.8%) in our study received 8 doses of Cresp^®^ at a dosage of 200 µg, with an average total dose per patient of 1474 µg, consistent with other studies where darbepoetin alfa demonstrated optimal outcomes in patients with CIA (16, 24).

ESAs have been found to be associated with higher risk of thromboembolic events in patients who have a Hb level > 10g/dL. Further, there is concern of stimulation of tumor growth with their use. A study by Henke et al., demonstrated that patients with carcinoma of head and neck had poorer locoregional progression-free survival if their tumor was positive for erythropoietin receptor expression and had been administered with epoetin beta for anemia (25). Based on this and other similar studies, the US FDA published a warning statement advising to restrict use of ESAs only to palliative anti-neoplastic therapy. Subsequently guidelines such as by ASH, ASCO, and the NCCN also recommended ESAs only when the aim of the therapy was palliative (4). A meta-analysis of RCTs involving ESAs in lung cancer patients revealed that ESA use resulted in higher incidence of thrombotic vascular adverse events but with no significant difference in mortality and adverse reactions when compared to control (placebo/no placebo). The study also reported reduced BT requirement with ESA use (26). Our study involved patients who were on a myelosuppressive therapy in a palliative setting who had an Hb level below 10.0 g/dL. Therefore, the eligibility criteria of our study aligns with the updated guidelines and recommendations of recognized organizations and societies.

In conclusion, Cresp^®^ emerges as an effective and well-tolerated intervention for patients with chemotherapy-induced anemia.

### 4.1 Drawbacks

As this is a prospective analysis of retrospectively gathered data, limitations exist in establishing a cause-effect relationship due to the nature of the study. Consequently, analyses such as duration to response could not be performed. A prospectively designed study would be ideal to measure such endpoints. Efforts have been made to prevent over generalization of the results. It is important to note that comorbidities within our patient cohort may differ from those in other Indian studies due to the initial manual data collection process which was later transferred into an electronic system, potentially introducing bias. Patient-reported outcome (PRO) data on fatigue, dyspnea, and headache were obtained using an in-house developed scale during morning rounds rather than through a standard questionnaire or guideline.

## 5 Conclusion

In conclusion, our retrospective study sheds light on the real–world effectiveness and tolerability of Cresp^®^ in addressing chemotherapy-induced anemia (CIA) in a real-world setting. The data suggests that Cresp^®^ effectively boosts hemoglobin levels, reducing the need for blood transfusions and improving the quality of life for cancer patients undergoing chemotherapy in a palliative setting. These findings provide practical insights that can assist clinicians in making informed decisions.

## Data Availability

The raw data supporting the conclusions of this article will be made available by the authors, without undue reservation.

## References

[1] Rodgers GM Becker PS Blinder MA Cella D Chanan-Khan A Cleeland CS. Cancer- and chemotherapy-induced anemia. J Natl Compr Cancer Network. (2012) 10:628–53. doi: 10.6004/jnccn.2012.0064 22570293

[2] Sathishkumar K Chaturvedi M Das P Stephen S Mathur P. Cancer incidence estimates for 2022 & projection for 2025: Result from National Cancer Registry Programme, India. Indian J Med Res. (2022) 156:598–607. doi: 10.4103/ijmr.ijmr_1821_22 36510887 PMC10231735

[3] Madeddu C Gramignano G Astara G Demontis R Sanna E Atzeni V. Pathogenesis and treatment options of cancer related anemia: Perspective for a Targeted Mechanism-Based Approach. Front Physiol. (2018) 9:1294. doi: 10.3389/fphys.2018.01294 30294279 PMC6159745

[4] Abdel-Razeq H Hashem H. Recent update in the pathogenesis and treatment of chemotherapy and cancer induced anemia. Crit Rev Oncol Hematol. (2020) 145:102837. doi: 10.1016/j.critrevonc.2019.102837 31830663

[5] Schoener B Borger J. Erythropoietin stimulating agents StatPearls - NCBI Bookshelf. (2023). Available at: https://www.ncbi.nlm.nih.gov/books/NBK536997/. 30725682

[6] Gilreath JA Rodgers GM. How I treat cancer-associated anemia. Blood. (2020) 136:801–13. doi: 10.1182/blood.2019004017 32556170

[7] Griffiths EA Roy V Alwan L Bachiashvili K Baird JH Cool R. NCCN guidelines^®^ Insights: hematopoietic growth factors, version 1.2022. J Natl Compr Cancer Network. (2022) 20:436–42. doi: 10.6004/jnccn.2022.0026 35545171

[8] Eisenhauer E Therasse P Bogaerts J Schwartz LH Sargent DJ Ford R. New response evaluation criteria in solid tumours: Revised RECIST guideline (version 1.1). Eur J Cancer. (2009) 45:228–47. doi: 10.1016/j.ejca.2008.10.026 19097774

[9] DelveInsight. Chemotherapy Induced anemia treatment, Companies, market trends. Chemotherapy Induced anemia infographic. Available online at: https://www.delveinsight.com/infographics/chemotherapy-induced-anemia-market (Accessed November 17, 2023).

[10] Khorana AA Francis CW Blumberg N Culakova E Refaai MA Lyman GH. Blood transfusions, thrombosis, and mortality in hospitalized patients with cancer. Arch Intern Med. (2008) 168:2377–81. doi: 10.1001/archinte.168.21.2377 PMC2775132 19029504

[11] Dasararaju R Marques MB. Adverse effects of transfusion. Cancer Control. (2015) 22:16–25. doi: 10.1177/107327481502200104 25504275

[12] Aapro M Béguin Y Bokemeyer C Dicato M Gascón P Glaspy JA. Management of anaemia and iron deficiency in patients with cancer: ESMO Clinical Practice Guidelines. Ann Oncol. (2018) 29:iv96–110. doi: 10.1093/annonc/mdx758 29471514

[13] Bokemeyer C Aapro M Courdi A Foubert J Link H Österborg A. EORTC guidelines for the use of erythropoietic proteins in anaemic patients with cancer: 2006 update. Eur J Cancer. (2007) 43:258–70. doi: 10.1016/j.ejca.2006.10.014 17182241

[14] Bohlius J Bohlke K Castelli R Djulbegoviĉ B Lustberg M Martino M. Management of cancer-associated anemia with erythropoiesis-stimulating agents: ASCO/ASH clinical practice guideline update. J Clin Oncol. (2019) 37:1336–51. doi: 10.1200/JCO.18.02142 30969847

[15] Grant MD Piper M Bohlius J Tonia T Robert N Vats V. Epoetin and Darbepoetin for Managing Anemia in Patients Undergoing Cancer Treatment: Comparative Effectiveness Update. Rockville, MD: Agency for Healthcare Research and Quality (US (2013). Available at: https://www.researchgate.net/publication/240043340_Epoetin_and_Darbepoetin_for_Managing_Anemia_in_Patients_Undergoing_Cancer_Treatment_Comparative_Effectiveness_Update (Accessed November 17, 2023). 23741758

[16] Gascón P Nagarkar R Šmakal M Syrigos K Barrios CH Sánchez JLGM. A randomized, Double-Blind, Placebo-Controlled, Phase III noninferiority study of the Long-Term Safety and Efficacy of Darbepoetin Alfa for Chemotherapy-Induced Anemia in patients with Advanced NSCLC. J Thorac Oncol. (2020) 15:190–202. doi: 10.1016/j.jtho.2019.10.005 31629060

[17] Steinmetz T Kindler M Lange O Vehling-Kaiser U Kuhn A Hellebrand E. A prospective cohort study on the impact of darbepoetin alfa on quality of life in daily practice following anemia treatment guideline revisions. Curr Med Res Opinion. (2014) 30:1813–20. doi: 10.1185/03007995.2014.924914 24849527

[18] Charu V Belani CP Gill AN Bhatt M Tomita D Rossi G. Efficacy and safety of every-2-week darbepoetin alfa in patients with anemia of cancer: A controlled, randomized, open-label phase II trial. Oncologist. (2007) 12:727–37. doi: 10.1634/theoncologist.12-6-727 17602062

[19] Vansteenkiste J Pirker R Massuti B Barata F Font A Fiegl M. Double-blind, placebo-controlled, randomized phase III trial of darbepoetin alfa in lung cancer patients receiving chemotherapy. J Natl Cancer Institute. (2002) 94:1211–20. doi: 10.1093/jnci/94.16.1211 12189224

[20] Glaspy JA Jadeja JS Justice G Kessler J Richards D Schwartzberg L. Darbepoetin alfa given every 1 or 2 weeks alleviates anaemia associated with cancer chemotherapy. Br J Cancer. (2002) 87:268–76. doi: 10.1038/sj.bjc.6600465 PMC2364226 12177793

[21] Vadhan-Raj S Mirtsching B Charu V Terry D Rossi G Tomita D. Assessment of hematologic effects and fatigue in cancer patients with chemotherapy-induced anemia given darbepoetin alfa every two weeks. J supportive Oncol. (2003) 1:131–8. Available at: https://utsouthwestern.elsevierpure.com/en/publications/assessment-of-hematologic-effects-and-fatigue-in-cancer-patients-. 15352656

[22] Vansteenkiste J Wauters I Elliott S Glaspy JA Hedenus M. Chemotherapy-induced anemia: the story of darbepoetin alfa. Curr Med Res Opinion. (2013) 29:325–37. doi: 10.1185/03007995.2013.766593 23323876

[23] Esquerdo G Llorca C Cervera JM Orts D Juárez A Carrato A. Effectiveness of darbepoetin alfa in a cohort of oncology patients with chemotherapy-induced anaemia. Relationship between variation in three fatigue-specific quality of life questionnaire scores and change in haemoglobin level. Clin Trans Oncol. (2011) 13:341–7. doi: 10.1007/s12094-011-0664-3 21596663

[24] Jagiełło-Gruszfeld A Niwińska A Michalski W Pogoda K Dubiański R Kunkiel M. Results of darbepoetin alfa treatment of anaemia in chemotherapy-receiving breast cancer patients: a single-centre retrospective observational study. Oncol Clin Practice. (2021) 17:237–43. doi: 10.5603/ocp.2020.0048

[25] Henke M Mattern D Pepe M Bézay C Weissenberger C Werner M. Do erythropoietin receptors on cancer cells explain unexpected clinical findings? J Clin Oncol. (2006) 24(29):4708–13. doi: 10.1200/JCO.2006.06.2737 17028293

[26] Tong Z Xu Z Duan Y Sun X Qu B. The effect of erythropoiesis−stimulating agents on lung cancer patients: a meta−analysis. Clin Exp Med. (2024) 24(1):150. doi: 10.1007/s10238-024-01391-3 38967734 PMC11226476

