# Supplementary material for: Cresp®: transforming the landscape of chemotherapy-induced anemia - a comprehensive retrospective real-world analysis in 523 Indian patients
Source: Front Oncol. 2025 Jan 24;15:1418327. doi: 10.3389/fonc.2025.1418327 (PMC11802802; doi:10.3389/fonc.2025.1418327)
Supplement: Supplementary file 1 [file Table1.docx]

**"Cresp®: Transforming the Landscape of Chemotherapy-Induced Anemia - A Comprehensive Retrospective Real-World Analysis in 523 Indian Patients"**

***Supplementary material***

Supplementary Table S1: Patient Reported Outcomes Scale

|  | 0 | 1 | 2 | 3 |
| --- | --- | --- | --- | --- |
| Dyspnea | - No physical limitation - Breathlessness only on strenuous activity | - Breathlessness when walking in fast pace at level ground - Limitation is usual day-to-day activities such as bathing, combing, and dressing. | - Breathlessness when walking at normal pace at level ground - Slight limitation in usual day-to-day activities such as bathing, combing, and dressing. | - Breathlessness when walking less than 100 meters - Marked limitations in usual day-to-day activities such as bathing, combing, and dressing. |
| Headache | - No pain | - Minor pain not requiring any use of analgesics - No limitation in independent functioning | - Noticeable pain, requiring occasional use of OTC analgesics - No or some limitation in independent functioning | - Severe pain, requiring regular use of analgesics - Pain needing visit to a healthcare professional - Marked limitation in independent functioning |
| Fatigue | - No fatigue | - Some tiredness - Able to carry out daily personal and professional activities independently | - Marked tiredness - Able to only essential activities | - Severe tiredness - Need assistance in carrying out even essential activities. |

Supplementary Table S2: Patient Characteristics

| **Demographics** | **N (%)** |
| --- | --- |
| Median age | 55 |
| Males | 264 (50.5) |
| Females | 259 (49.5) |
| **Comorbidities** | |
| Hypertension | 93 (34.3) |
| Diabetes mellitus | 48 (17.7) |
| Chronic obstructive pulmonary disease | 44 (16.2) |
| Chronic liver disease | 26 (9.6) |
| Hypothyroidism | 22 (8.1) |
| Coronary artery disease | 20 (7.4) |
| Chronic kidney disease | 18 (6.7) |
| **Cancer site** |  |
| Gastrointestinal | 167 (31.9) |
| Breast | 85 (16.3) |
| Gynaecological | 79 (15.1) |
| Genitourinary | 58 (11.1) |
| Lung | 55 (10.5) |
| Head & Neck Squamous Cell Carcinoma | 33 (6.3) |
| Others | 46 (8.8) |
| **Chemotherapy agents** |  |
| Single – agent | 133 (25.4) |
| Multiple – agents | 390 (74.6) |
| **Chemotherapy types** |  |
| Taxane + Platinum | 120 (22.9) |
| Gemcitabine + Platinum | 95 (18.2) |
| Fluoropyrimidine – based | 86 (16.5) |
| Single – Agent Taxane | 67 (12.8) |
| Platinum + Other Agent/ Platinum Mono | 41 (7.8) |
| Taxane + Other Agent | 33 (6.3) |
| Pemetrexed/Methotrexate – based | 23 (4.4) |
| Anthracycline – based | 17 (3.3) |
| Others | 41 (7.8) |
| **Use of Platinum agents** |  |
| Platinum – regimen | 354 (67.7) |
| Non – platinum regimen | 169 (32.3) |
| **Response to therapy** |  |
| Partial response | 307 (58.7) |
| Stable disease | 159 (30.4) |
| Progression of disease | 57 (10.9) |
| **No. of doses of Darbepoetin alfa** |  |
| 4 Doses | 59 (11.3) |
| 5 Doses | 22 (4.2) |
| 6 Doses | 14 (2.7) |
| 8 Doses | 428 (81.8) |
| **Hemoglobin level at baseline** |  |
| <8 g/dl | 1 (0.2) |
| 8 – 10 g/dl | 515 (98.5) |
| >10 and ≤ 11 g/dl | 7 (1.3) |
| >11 g/dl | 0 (0.0) |
| **Hemoglobin level at end of treatment** |  |
| <8 g/dl | 0 (0.0) |
| 8 – 10 g/dl | 82 (15.7) |
| >10 and ≤ 11 g/dl | 209 (39.9) |
| >11 g/dl | 232 (44.4) |

Supplementary Table S3: 29 different malignancies grouped into 7 major Cancer types

| Cancer Type (7) | Malignancies (29) |
| --- | --- |
| Gastrointestinal | Anal canal, biliary tract, colon, duodenal, esophagus, pancreas, stomach (gastroesophageal junction) |
| HNSCC | Head and neck squamous cell carcinoma, nasopharynx, salivary gland |
| Lung | Non-small cell lung cancer, small cell lung cancer, mesothelioma |
| Breast | Breast |
| Genitourinary | Germ cell tumor, penile, prostate, urachus, urothelial |
| Gynecological | Cervix, ovary, uterus |
| Others | Adrenal cortex, carcinoma of unknown primary, melanoma, neuroendocrine, sarcoma, skin squamous cell carcinoma, thyroid |

Supplementary Table S4: 81 different chemotherapy regimens were grouped into 9 major Chemotherapy types

| Types | Fluoropyrimidine-Based | Taxane + Platinum | Gemcitabine + Platinum | Single-Agent Taxane | Pemetrexed/ Methotrexate – Based | Anthracycline-Based | Taxane + Other Agent | Platinum + Other Agent/ Platinum Mono | Others |
| --- | --- | --- | --- | --- | --- | --- | --- | --- | --- |
| Regimen | FOLFIRI | PACLI CARBO | GEM CIS | PACLI | PEME CIS | ETO CIS DOXO | GEM DOCE | IRI CARBO | IRI |
|  | DOCE CIS 5FU | PACLI CARBO TRASTU | GEM OX | DOCE | PEME CARBO GEFITINIB | ADRIA CYCLO | PACLI TRASTU | CIS IRI BEV | IFO EPI |
|  | FOLFOX BEV | DOCE CARBO | GEM CARBO |  | PEME | DOXO BEV | PACLI TRASTU PERTU | CIS NIMOTO | OGS |
|  | FOLFIRINOX | DOCE CIS | GEM CARBO TRASTU |  | PEME GEF | DOXO CARBO | DOCE CYCLO | PEMBRO CARBO BEV | VIN ACTIN CYCLO |
|  | FOLFOX | PACLI CARBO BEV | GEM CIS BEV |  | PEME CARBO | DOXO CARBO BEV | PACLI TAMOXI TRASTU | TEMO CIS | VIME/VAC |
|  | FOLFOX PANITU | PACLI CIS BEV | GEM CARBO BEV |  | PEME BEV | VIN DOXO CYCLO | PACLI CETUXI | CARBO BEV | OGS 2012 |
|  | FOLFIRI PANITU | PACLI CARBO BEV ATEZO | GEM CIS TRASTU |  | EMACO | IFO ADRIA | DOCE TRASTU | CAPE OX | TEMO IRI |
|  | FOLFIRI BEV | PACLI CARBO PEMBRO | GEM CIS PEMBRO |  | VINBLA MTX TAMOXI |  | PACLI RAMU | ETO CARBO | VIME |
|  | FOLFIRI NIV | PACLI CARBO CETUXI |  |  | IFO EPI MTX |  |  | ETO CIS | GEM |
|  | DOCE OX 5FU | PACLI CIS BEV |  |  |  |  |  | ETO CIS DURVA |  |
|  | FOLFIRI RAMU | PACLI CARBO TRASTU |  |  |  |  |  | CABAZI CARBO |  |
|  | 5FU |  |  |  |  |  |  | CARBO |  |
|  | 5FU TRASTU |  |  |  |  |  |  |  |  |
|  | FLOT |  |  |  |  |  |  |  |  |
|  | PACLI CARBO 5FU |  |  |  |  |  |  |  |  |

Supplementary Table S5: Chemotherapy abbreviations:

| 5FU | 5-Fluorouracil |
| --- | --- |
| ADRIA | Adriamycin |
| ATEZO | Atezolizumab |
| BEV | Bevacizumab |
| CABAZI | Cabazitaxel |
| CAPE OX | Capecitabine Oxaliplatin |
| CARBO | Carboplatin |
| CETUXI | Cetuximab |
| CIS | Cisplatin |
| CYCLO | Cyclophosphamide |
| DOCE | Docetaxel |
| DOXO | Doxorubicin |
| DURVA | Durvalumab |
| EMACO | Etoposide, Methotrexate, Dactinomycin, Cyclophosphamide, Vincristine |
| EPI | Epirubicin |
| ETO | Etoposide |
| FOLFIRI | Folinic Acid+ 5 Fluorouracil + Irinotecan |
| FULV | Fulvestrant |
| GEF | Gefitinib |
| GEM | Gemcitabine |
| GNRH | Gonadotropin Releasing Hormone Agonist |
| IFO | Ifosfamide |
| IRI | Irinotecan |
| NIMOTO | Nimotuzumab |
| OGS | Doxorubicin, Cisplatin and Ifosfamide |
| OSI | Osimertinib |
| PACLI | Paclitaxel |
| PANITU | Panitumumab |
| PEMBRO | Pembrolizumab |
| PEME | Pemetrexed |
| PERTU | Pertuzumab |
| RAMU | Ramucirumab |
| TAMOXI | Tamoxifen |
| TDM1 | Trastuzumab Emtansine |
| TEMO | Temozolomide |
| TRASTU | Trastuzumab |
| VIME | Vincristine, Ifosfamide, Mesna, Etoposide |
| VIN | Vincristine |
| VINBLA | Vinblastine |
| VINOREL | Vinorelbine |

Supplementary Table S6: Platinum agents:

| Carboplatin |
| --- |
| Cisplatin |
| Oxaliplatin |

Supplementary Table S7: Sub-group analysis for mean haemoglobin increment in each sub-group

| **Cancer site** | **N (%)** | **Hb Increment (Mean ± SD)** |
| --- | --- | --- |
| Gastrointestinal | 167 (31.9) | 2.38 ± 0.97 |
| Breast | 85 (16.3) | 2.29 ± 0.62 |
| Gynaecological | 79 (15.1) | 2.19 ± 0.69 |
| Genitourinary | 58 (11.1) | 2.17 ± 0.72 |
| Lung | 55 (10.5) | 2.36 ± 0.52 |
| Head & Neck Squamous Cell Carcinoma | 33 (6.3) | 2.38 ± 0.74 |
| Others | 46 (8.8) | 2.00 ± 0.76 |
| **Chemotherapy regimen** | | |
| Single – agent | 133 (25.4) | 2.35 ± 0.76 |
| Multiple – agent | 390 (74.6) | 2.25 ± 0.79 |
| **Chemotherapy types** | | |
| Taxane + Platinum | 120 (22.9) | 2.26 ± 0.67 |
| Gemcitabine + Platinum | 95 (18.2) | 2.30 ± 0.93 |
| Fluoropyrimidine - based | 86 (16.5) | 2.31 ± 0.85 |
| Single-Agent Taxane | 67 (12.8) | 2.39 ± 0.71 |
| Platinum + Other Agent/ Platinum Mono | 41 (7.8) | 2.12 ± 0.86 |
| Taxane + Other Agent | 33 (6.3) | 2.49 ± 0.64 |
| Pemetrexed/Methotrexate -based | 23 (4.4) | 2.49 ± 0.65 |
| Anthracycline - based | 17 (3.3) | 1.95 ± 0.88 |
| Others | 41 (7.8) | 2.03 ± 0.68 |
| **Use of Platinum agents** | | |
| Platinum | 354 (67.7) | 2.27 ± 0.82 |
| Non-platinum | 169 (32.3) | 2.29 ± 0.71 |
| **Response to therapy*** | | |
| Partial response | 307 (58.7) | 2.66 ± 0.51 |
| Stable disease | 159 (30.4) | 1.86 ± 0.82 |
| Progression of disease | 57 (10.9) | 1.40 ± 0.59 |
| **No. of doses of Darbepoetin alfa*** | | |
| 4 Doses | 59 (11.3) | 1.61 ± 0.67 |
| 5 Doses | 22 (4.2) | 2.26 ± 0.54 |
| 6 Doses | 14 (2.7) | 2.54 ± 1.11 |
| 8 Doses | 428 (81.8) | 2.36 ± 0.76 |

**P<0.001, one-way ANOVA*
